# High multidrug resistance in urinary tract infections in a tertiary hospital, Kathmandu, Nepal

**DOI:** 10.5588/pha.21.0035

**Published:** 2021-11-01

**Authors:** S. Shakya, J. Edwards, H. A. Gupte, S. Shrestha, B. M. Shakya, K. Parajuli, H. P. Kattel, P. S. Shrestha, R. Ghimire, P. Thekkur

**Affiliations:** 1 Central Department of Public Health, Tribhuvan University, Institute of Medicine, Kathmandu, Nepal; 2 Department of Global Health, University of Washington, Seattle, WA, USA; 3 Narotam Sekhsaria Foundation, Mumbai, India; 4 World Health Emergencies Programme, WHO Country Office, Kathmandu, Nepal; 5 Department of Anaesthesiology, Maharajgunj Medical Campus, Tribhuvan University, Institute of Medicine, Kathmandu, Nepal; 6 Department of Clinical Microbiology, Tribhuvan University Teaching Hospital, Kathmandu, Nepal; 7 Department of Clinical Pharmacology, Maharajgunj Medical Campus, Tribhuvan University, Institute of Medicine, Kathmandu, Nepal; 8 Centre for Operational Research, International Union Against Tuberculosis and Lung Disease (The Union), Paris, France; 9 Centre for Operational Research, The Union South-East Asia Office, New Delhi, India

**Keywords:** SORT IT, AMR, presumptive UTI, bacteriological profile, drug susceptibility

## Abstract

**SETTING:**

Tribhuvan University Teaching Tertiary Care Hospital, Kathmandu, Nepal, May–October 2019.

**OBJECTIVE:**

1) To describe the bacteriological profile, 2) to identify the antimicrobial resistance (AMR) pattern, and 3) to find the demographic characteristics associated with the presence of bacterial growth and multidrug resistance (MDR) in adult urine samples undergoing culture and drug susceptibility testing.

**DESIGN:**

This was a hospital-based, cross-sectional study using routine laboratory records.

**RESULTS:**

Among 11,776 urine samples, 16% (1,865/11,776) were culture-positive, predominantly caused by *Escherichia coli* (1,159/1,865; 62%). We found a high prevalence of resistance to at least one antibiotic (1,573/1,865; 84%) and MDR (1,000/1,865; 54%). Resistance to commonly used antibiotics for urinary tract infections (UTIs) such as ceftazidime, levofloxacin, cefepime and ampicillin was high. Patients aged ⩾60 years (adjusted prevalence ratio [aPR] 1.6, 95% CI 1.4–1.7) were more likely to have culture positivity. Patients with age ⩾45 years (45–59 years: aPR 1.5, 95% CI 1.3–1.7; ⩾60 years: aPR 1.4, 95% CI 1.2–1.6), male sex (aPR 1.3, 95% CI 1.2–1.5) and from inpatient settings (aPR 1.4, 95% CI 1.2–1.7) had significantly higher prevalence of MDR.

**CONCLUSION:**

Urine samples from a tertiary hospital showed high prevalence of *E. coli* and MDR to routinely used antibiotics, especially among inpatients. Regular surveillance and application of updated antibiograms are crucial to monitor the AMR situation in Nepal.

## Abstract (FR)

**LIEU:**

Hôpital universitaire de soins tertiaires de Tribhuvan, Katmandu, Népal, mai–octobre 2019.

**OBJECTIF:**

1) Décrire le profil bactériologique, 2) identifier le profil de résistance antimicrobienne (AMR), et 3) identifier les caractéristiques démographiques associées à la présence de croissance bactérienne et de résistance à plusieurs médicaments (MDR) dans les échantillons urinaires d’adultes mis en culture et testés pour sensibilité aux médicaments.

**MÉTHODE:**

Il s’agissait d’une étude transversale hospitalière réalisée en utilisant les dossiers de laboratoire de routine.

**RÉSULTATS:**

Parmi 11 776 échantillons urinaires, 16% (1 865/11 776) étaient positifs par culture, principalement à *Escherichia coli* (1 159/1 865 ; 62%). Nous avons observé une prévalence élevée de résistance à au moins un antibiotique (1 573/1 865 ; 84%) et de MDR (1 000/1 865 ; 54%). La résistance aux antibiotiques fréquemment utilisés dans le traitement des infections urinaires (UTI), comme la ceftazidime, la lévofloxacine, la céfépime et l’ampicilline était élevée. Les patients âgés ⩾ 60 ans (ratio de prévalence ajusté [aPR] 1,6 ; IC 95% 1,4–1,7) étaient plus susceptibles d’avoir une culture positive. Les patients âgés de ⩾ 45 ans (45–59 ans : aPR 1,5 ; IC 95% 1,3–1,7 ; ⩾ 60 ans : aPR 1,4 ; IC 95% 1,2–1,6), les hommes (aPR 1,3 ; IC 95% 1,2–1,5) et les patients hospitalisés (aPR 1,4 ; IC 95% 1,2–1,7) avaient une prévalence significativement plus élevée de MDR.

**CONCLUSION:**

Les échantillons urinaires d’un hôpital tertiaire étaient associés à une prévalence élevée d’*E. coli* et de MDR aux antibiotiques utilisés en routine, notamment chez les patients hospitalisés. Une surveillance régulière et l’utilisation d’antibiogrammes à jour sont essentielles au suivi de l’AMR au Népal.

Urinary tract infections (UTIs) are one of the leading causes of morbidity and growing health care expenditure worldwide.^1^ These are the most common bacterial infections seen in tertiary care hospitals, with higher morbidity and mortality among developing countries.^2^,^3^ The WHO has reported *Escherichia coli* and *Klebsiella pneumoniae* as the most common bacteria causing UTIs.^4^ The burden of UTIs worldwide leads to increased antibiotic usage, including both self-administration and inappropriate prescribing.^2^,^5^ Although about 80% of those with UTI are managed in outpatient departments,^6^ inappropriate empirical therapy is associated with prolonged treatments, hospital stays, increased costs and higher mortality.^7^,^8^ UTI prevalence among Nepalese patients attending general hospitals ranges from 23% to 37%.^9^

Antimicrobial resistance (AMR) is a rapidly emerging problem, especially in low and middle-income countries (LMICs) and urinary pathogens are among the most frequently resistant.^10^,^11^ The most common urinary pathogen in Europe, *E. coli* has a reported multidrug resistance (MDR) rate of 15%.^12^ MDR has been reported to be significantly higher in LMICs.^10^ Studies in Asia Pacific regions show higher AMR prevalence in different categories of antibiotics used for the treatment of UTIs.^13^ A study conducted in 2019 from Nepal found the MDR of *E. coli* and *K. pneumoniae* among hospitalised patients with UTIs to be 62%.^14^ The direct consequences of AMR include prolonged illness and hospital stay, mortality and increased costs. Furthermore, AMR will most likely impact achievement of the Sustainable Development Goal 3, which aims to ‘ensure healthy lives and promote well-being for all at all ages’.^15^ However, the indirect impact extends beyond public health and has been linked to adversely affecting development and the global economy.^8^

The WHO has focused on a lack of systematic data collection on AMR in the South-East Asia Region (SEAR), and described the AMR problem as being ‘burgeoning and often neglected’.^4^ In response to AMR being a pivotal worldwide healthcare challenge, the WHO has developed the Global Action Plan on AMR (GAP-AMR) and the Global Antimicrobial Resistance Surveillance System (GLASS) in 2015.^8^

Nepal is still in the process of implementing the five WHO strategies for tackling AMR through the endorsement of a national action plan to combat the growing AMR crisis. Unfortunately, there is lack of reliable information within the SEAR, particularly Nepal, where AMR has become a crucial issue.^16^,^17^ Due to the increased frequency of AMR among UTIs and related worse outcomes in LMICs, there is an urgent need to have an improved understanding of the situation.

Keeping in mind two strategic objectives of the WHO, 1) strengthening the knowledge and evidence base through surveillance and research, and 2) optimising antibiotic use through stewardship and surveillance, this study aimed to identify the pattern of AMR among adult urine samples undergoing culture and drug susceptibility testing (CDST) in a tertiary hospital of Kathmandu from May to October 2019. The specific objectives were to 1) describe the demographic profile of the patients who underwent urine CDST; 2) describe the bacteriological profile and corresponding AMR pattern; and 3) find demographic characteristics associated with the presence of bacterial growth and MDR.

## METHODS

### Study design

This was a hospital-based, cross-sectional study involving review of previously collected routine laboratory records.

### Setting

The study setting was Tribhuvan University Teaching Hospital (TUTH), Kathmandu, Nepal, which is the first teaching hospital of the country, established in 1983. TUTH is a comprehensive public, tertiary-care, referral, 700-bed facility, with both outpatient and inpatient departments including an intensive care unit, and emergency, maternal-child health, medical, surgical and other subspecialty departments.

### Laboratory services

The hospital has a centralised laboratory, including microbiology services. The Microbiology Department collects all urine specimens for CDST, which are then sent to the laboratory for CDST for those patients with symptoms of UTI, fever, presence of pus cells (>2 for males and ⩾4 for females) in urine routine examination, pregnant women (for diagnosis of asymptomatic bacteriuria) and patients who are under urinary catheterisation for a long time. Generally, the report of urine CDST is available to the patients in 24–48 hours. While waiting for the culture report, empirical treatment with first-line antibiotics is initiated.

### CDST protocol

As per standardised protocol, clean-catch midstream urine is collected in a sterile container. For patients with indwelling urinary catheter, the tube is clamped for several minutes before the sample is drawn from the tube. The samples are immediately sent to the laboratory and are inoculated on blood agar, MacConkey’s agar and cystine–lactose–electrolyte-deficient (CLED) agar plates using flame sterilised nichrome wire loop (internal diameter of 4 mm holding 0.01ml).

A semi-quantitative method is utilised for urine cultures. The plates are incubated at 35°C and are observed for bacterial growth after 24 h. The bacteria are identified according to colony characteristics, Gram’s staining and biochemical properties. Bacterial colonies more than 105 colony-forming units (CFU) per ml of urine are generally considered to represent significant bacteriuria. These are then subjected to antibiogram testing by Kirby-Bauer’s disc diffusion method using Mueller-Hinton agar for identifying bacterial susceptibility and resistance.^18^

### Study population

The study population included all urine samples submitted from inpatients and outpatients, who were aged >18 years, were attending TUTH and undergoing urine CDST from 1 May to 31 October 2019 (6-month period).

### Data variables, sources and collection

Data of patients who underwent urine CDST from May to October 2019 were extracted from the laboratory registers. Data variables included date of specimen sent to laboratory, status of patient (inpatient/outpatient), age, sex, department, culture growth, bacteria isolated in culture and antibiotic resistance pattern (susceptible/resistant) to any antibiotic.

### Data analysis

Data were entered using EpiData Entry software v3.1 (EpiData Association, Odense, Denmark). This was manually cross-checked, edited and cleaned for data entry errors. Data were analysed using Stata v12 (StataCorp, College Station, TX, USA). The demographic details of the presumptive UTI patients, the bacteriological profile of patients with culture-positive urine and the AMR pattern were summarised using numbers and proportions. The isolates with resistance to at least one drug in three or more classes of antibiotics was classified as multidrug-resistant.^19^ The association of demographic characteristics with presence of bacterial growth and MDR was assessed using modified Poisson regression with variance robust estimates (univariate and also multivariate). The prevalence ratio (PR) and adjusted prevalence ratio (aPR) with 95% confidence interval (CI) were used as a measure of association in the univariate and the multivariate models.

### Ethical approval

Ethical approval was obtained from the Union Ethics Advisory Group, the International Union Against Tuberculosis and Lung Disease, Paris, France (EAG 09/20); and the Institutional Review Committee, Tribhuvan University, Institute of Medicine, Kathmandu, Nepal [314(6-11)E^2^076/077].

## RESULTS

Of a total of 11,776 adult samples that underwent urine CDST, 8,660 (73.5%) were outpatients (Figure, Table 1). Most samples were from patients aged 18–29 years (4,063/11,776; 34.5%) and were more frequently from females in both the outpatient (5,498/8,660; 63.5%) and inpatient (2,397/3,116; 76.9%) settings. During the study period, nearly one fifth (2,278/11,776; 19.3%) of the samples underwent urine culture during August.

**FIGURE:**
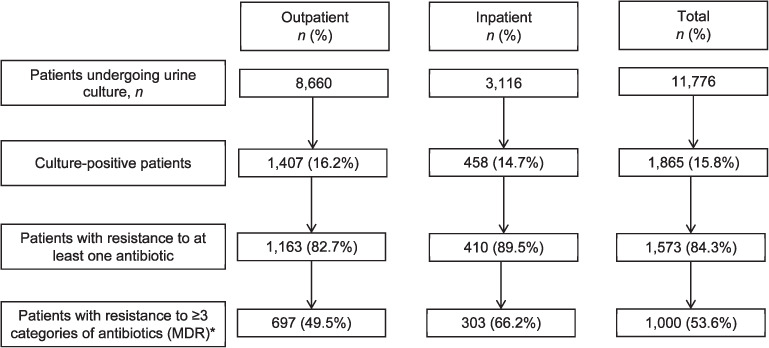
Flow chart of urine culture results and antibiotic resistance among adult samples undergoing urine culture and drug susceptibility testing in Kathmandu, Nepal, May–October 2019. ^*^The percentage was calculated based on the number of culture-positive individuals as denominator.

**TABLE 1:** Demographic characteristics of adult samples undergoing urine culture and drug susceptibility test in Kathmandu, Nepal, May–October 2019 (n = 11,776)

Characteristics	Inpatient		Outpatient		Total	
		
	*n*	(%)^*^	*n*	(%)^*^	*n*	(%)^*^
Total	3,116	(26.5)	8,660	(73.5)	11,776	(100.0)
Age, years						
18–29	1,312	(42.1)	2,751	(31.8)	4,063	(34.5)
30–44	853	(27.4)	2,322	(26.8)	3,175	(27.0)
45–59	419	(13.4)	1,511	(17.4)	1,930	(16.4)
⩾60	532	(17.1)	2,076	(24.0)	2,608	(22.1)
Sex						
Male	718	(23.0)	3,104	(35.8)	3,822	(32.5)
Female	2,397	(76.9)	5,498	(63.5)	7,895	(67.0)
Not recorded	1	(0.0)	58	(0.7)	60	(0.5)
Department						
Medicine	154	(4.9)	17	(0.2)	171	(1.5)
Surgery	741	(23.8)	35	(0.4)	776	(6.6)
Obstetrics/Gynaecology	759	(24.4)	14	(0.2)	773	(6.6)
Nephrology	158	(5.1)	6	(0.1)	164	(1.4)
Others^†^	1,113	(35.8)	15	(0.2)	1,128	(9.6)
Not recorded	191	(6.1)	8,573	(99.0)	8,764	(74.4)
Month of testing						
May	525	(16.8)	1,278	(14.8)	1,803	(15.3)
June	509	(16.3)	1,479	(17.1)	1,988	(16.9)
July	570	(18.3)	1,509	(17.4)	2,079	(17.7)
August	614	(19.7)	1,664	(19.2)	2,278	(19.3)
September	504	(16.2)	1,624	(18.8)	2,128	(18.1)
October	394	(12.6)	1,106	(12.8)	1,500	(12.7)

^*^ Column percentage.

^†^ Include Orthopaedics; Ear, Nose, Throat; Psychiatry; Burn Ward; Intensive Care Unit.

Of the 11,776 samples undergoing urine culture test, 15.8% (1,865/11,776) were culture-positive for bacterial isolate: 16.2% (1,407/8,660) were positive among outpatients and 14.7% (458/3,116) among in-patients. Of the 1,865 with confirmed infection, 84.3% (1,573/1,865) showed resistance to at least one antibiotic and 53.6% (1,000/1,865) had MDR. The proportion of MDR among isolates from outpatients and in-patients were respectively 49.5% (697/1,407) and 66.2% (303/458) (Figure).

*E. coli* was the most common organism found (1,159/1,865; 62.1%), followed by *K. pneumoniae* (191/1,865; 10.2%) and *Enterococcus* (184/1,865; 9.9%). Among outpatients, *E. coli* was the causative pathogen in the majority (952/1,407; 67.7%); there was a more diverse group of pathogens among inpatients (Table 2).

**TABLE 2:** Bacterial profile of adult samples with positive urine culture for bacterial isolate in Kathmandu, Nepal, May–October 2019

Organism	Inpatient		Outpatient		Total	
		
	*n*	(%)^*^	*n*	(%)^*^	*n*	(%)^*^
Total	458	(24.6)	1407	(75.4)	1865	(100.0)
*Escherichia coli*	207	(45.2)	952	(67.7)	1159	(62.1)
*Klebsiella pneumonia*	52	(11.4)	139	(9.9)	191	(10.2)
*Enterococcus*	81	(17.7)	103	(7.3)	184	(9.9)
*Pseudomonas aeruginosa*	68	(14.9)	95	(6.8)	163	(8.7)
*Staphylococcus aureus*	11	(2.4)	54	(3.8)	65	(3.5)
*Acenetobacter baumannii*	28	(6.1)	23	(1.6)	51	(2.7)
Others^†^	37	(8.1)	75	(5.3)	112	(6.0)

^*^ Column percentage.

^†^ Includes Citobacter species, Burkholderia, coagulase-negative Staphylococci, Enterobacter, Providencia.

Table 3 shows the resistance pattern found among Gram-negative bacterial isolates. There were 1,159 infections secondary to *E. coli*, the highest antibiotic resistance was to ceftazidime (125/151; 82.8%), levofloxacin (130/169; 76.9%) and ampicillin (864/1,147; 75.3%). There were 191 infections with *K. pneumoniae*; the highest antibiotic resistance among routinely used medications were to ceftazidime (72/75; 96.0%), cefepime (55/66; 83.3%) and levofloxacin (61/76; 80.3%). There were 163 cases of infection secondary to *Pseudomonas aeruginosa* with a significant amount of resistance to ciprofloxacin (83/156; 53.2%), gentamycin (70/155; 45.2%) and ceftazidime (59/151; 39.1%). Finally, there were 51 infections related to *Acinetobacter baumannii* with the highest resistance to nitrofurantoin (38/42; 90.5%), doxycycline (14/14; 100.0%) and ceftazidime (13/17; 76.5%). Moreover, there was resistance to meropenem (9/16; 56.3%) and imipenem (9/17; 52.9%), but no resistance to polymyxin B.

**TABLE 3:** Drug susceptibility testing and drug resistance patterns of common Gram-negative organisms detected among adult samples with positive urine culture for bacterial isolate in Kathmandu, Nepal, May–October 2019

Drugs	*Escherichia coli* (*n* = 1159)			*Klebsiella pneumoniae* (*n* =191)			*Pseudomonas aeruginosa* (*n* =163)			*Acenetobacter baumannii* (*n* = 51)		
			
	Test *N*	Resistant		Test *N*	Resistant		Test *N*	Resistant		Test *N*	Resistant	
			
		*n*	(%)^*^		*n*	(%)^*^		*n*	(%)^*^		*n*	(%)^*^
Amikacin	216	48	(22.2)	78	45	(57.7)	153	64	(41.8)	20	10	(50.0)
Amoxicillin clavulanate	1070	522	(48.8)	170	102	(60.0)	—	—	—	—	—	—
Amoxicillin/ampicillin	1147	864	(75.3)	—	—	—	—	—	—	—	—	—
Ampicillin-sulbactam	130	47	(36.2)	58	38	(65.5)	—	—	—	17	3	(17.7)
Cefoperazone-sulbactam	141	56	(39.7)	73	51	(69.9)	45	23	(51.1)	16	6	(37.5)
Cefepime	167	111	(66.5)	66	55	(83.3)	48	28	(58.3)	17	12	(70.6)
Cefixime/ceftriaxone	1112	643	(57.8)	185	98	(53.0)	—	—	—	47	30	(63.8)
Ceftazidime	151	125	(82.8)	75	72	(96.0)	151	59	(39.1)	17	13	(76.5)
Chloramphenicol	142	55	(38.7)	69	41	(59.4)	—	—	—	—	—	—
Colistin sulphate	134	0	(0.0)	71	0	(0.0)	40	0	(0.0)	16	0	(0.0)
Ciprofloxacin	598	330	(55.2)	117	60	(51.3)	156	83	(53.2)	21	10	(47.6)
Cotrimoxazole	1045	549	(52.5)	176	96	(54.6)	—	—	—	46	17	(37.1)
Doxycycline	144	91	(63.2)	71	56	(78.9)	—	—	—	14	14	(100.0)
Gentamycin	1108	146	(13.2)	185	56	(30.3)	155	70	(45.2)	50	17	(34.0)
Imipenem	151	22	(14.6)	71	33	(46.5)	49	28	(57.1)	17	9	(52.9)
Levofloxacin	169	130	(76.9)	76	61	(80.3)	153	78	(51.0)	18	8	(44.4)
Meropenem	148	24	(16.2)	69	36	(52.2)	49	28	(57.1)	16	9	(56.3)
Nitrofurantoin	1099	107	(9.7)	168	103	(61.3)	—	—	—	42	38	(90.5)
Norfloxacin	633	351	(55.5)	87	36	(41.4)	4	2	(50.0)	32	13	(40.6)
Piperacillin-tazobactam	1055	153	(14.5)	170	48	(28.2)	159	14	(8.8)	49	13	(26.5)
Polymyxin B	145	0	(0.0)	73	0	(0.0)	46	0	(0.0)	18	0	(0.0)
Aztreonam	1	1	(100)	—	—	—	1	0	(0.0)	—	—	—

^*^Column percentage.

Antibiotic resistance of Gram-positive bacterial isolates is shown in Table 4. There were 184 infections caused by *Enterococcus* and commonly showed resistance to amoxicillin (81/182; 44.5%), nitrofurantoin (44/166; 26.5%) and vancomycin (4/174; 2.3%). Likewise, 65 *Staphylococcus aureus* isolates detected were commonly resistant to amoxicillin/ampicillin (19/24; 79.2%), cotrimoxazole (15/54; 27.8%) and ciprofloxacin (18/55; 32.7%). There was no resistance found with amoxicillin-clavulanate.

**TABLE 4:** Drug susceptibility testing and drug resistance patterns of common Gram-positive organisms detected among adult samples with positive urine culture for bacterial isolate in Kathmandu, Nepal, May–October 2019

Drugs	*Enterococcus* (*n* = 184)			*Staphylococcus aureus* (*n* = 65)		
	
	Test *N*	Resistant		Test *N*	Resistant	
	
		*n*	(%)^*^		*n*	(%)^*^
Amikacin	6	5	(83.3)	9	1	(11.1)
Amoxicillin clavulanate	162	69	(42.6)	1	0	(0.0)
Amoxicillin/ampicillin	182	81	(44.5)	24	19	(79.2)
Ampicillin-sulbactam	1	0	(0.0)	1	0	(0.0)
Cefoperazone-sulbactam	—	—	—	1	0	(0.0)
Cefepime	—	—	—	1	0	(0.0)
Cefixime/ceftriaxone	—	—	—	3	1	(33.3)
Ceftazidime	—	—	—	1	0	(0.0)
Chloramphenicol	144	12	(8.3)	11	1	(9.1)
Ciprofloxacin	141	102	(72.3)	55	18	(32.7)
Cotrimoxazole	—	—	—	54	15	(27.8)
Doxycycline	143	120	(83.9)	4	0	(0.0)
Gentamycin	160	90	(56.3)	56	6	(10.7)
Imipenem	2	1	(50.0)	1	0	(0.0)
Levofloxacin	164	110	(67.1)	9	2	(22.2)
Meropenem	20	15	(75.0)	1	0	(0.0)
Nitrofurantoin	166	44	(26.5)	61	2	(3.3)
Cephalexin	—	—	—	57	8	(14.0)
Norfloxacin	64	51	(79.7)	—	—	—
Piperacillin-tazobactam	159	75	(47.2)	—	—	—
Vancomycin	174	4	(2.3)	—	—	—
Teicoplanin	170	2	(1.2)	—	—	—

^*^Column percentage.

Compared to samples from patients aged 18–29 years, those aged 45–59 years (aPR 1.3, 95% CI 1.2–1.5) and those aged ⩾60 years (aPR 1.6, 95% CI 1.4–1.7) had significantly higher rates of culture positivity (Table 5). Although isolates from outpatients (16.3%) showed higher culture positivity rates than those from inpatients (14.7%), there was no significant difference overall when compared.

**TABLE 5:** Demographic characteristics associated with presence of bacterial growth among adult samples undergoing urine culture and susceptibility test in Kathmandu, Nepal, May–October 2019 (n = 11,776)

Characteristics	Total *n*	Bacteria present		PR	(95% CI)	aPR	(95% CI)	*P* value

		*n*	(%)^*^					
Total	11,776	1865	(15.8)					
Age, years								
18–29	4,063	544	(13.4)	Reference		Reference		
30–44	3,175	420	(13.2)	1.0	(0.9–1.1)	1.0	(0.9–1.1)	0.668
45–59	1,930	349	(18.1)	1.4	(1.2–1.5)	1.3	(1.2–1.5)	<0.001
⩾60	2,608	552	(21.2)	1.6	(1.4–1.8)	1.6	(1.4–1.7)	<0.001
Sex								
Male	3,822	640	(16.8)	1.1	(1.0–1.2)	0.9	(0.9–1.0)	0.231
Female	7,895	1,216	(15.4)	1		Reference		
Not recorded	59	9	(15.3)	1.0	(0.5–1.8)	0.7	(0.4–1.4)	0.344
Department								
Medicine	171	32	(18.7)	1.8	(1.2–2.6)	1.5	(1.0–2.1)	0.052
Surgery	776	127	(16.4)	1.6	(1.2–2.0)	1.4	(1.0–1.8)	0.021
Obstetrics/Gynaecology	773	81	(10.5)	Reference		Reference		
Nephrology	164	31	(18.9)	1.8	(1.2–2.6)	1.5	(1.0–2.2)	0.031
Others^†^	1,128	167	(14.8)	1.4	(1.1–1.8)	1.2	(1.0–1.6)	0.087
Not recorded	8,764	1,427	(16.3)	1.6	(1.3–1.9)	1.5	(1.0–2.0)	0.028
Month of referral								
May	1,803	274	(15.2)	Reference		Reference		
June	1,988	286	(14.4)	0.9	(0.8–1.1)	0.9	(0.8–1.1)	0.489
July	2,079	308	(14.8)	1.0	(0.8–1.1)	1.0	(0.8–1.1)	0.680
August	2,278	365	(16.0)	1.1	(0.9–1.2)	1.0	(0.9–1.2)	0.589
September	2,128	370	(17.4)	1.1	(1.0–1.3)	1.1	(1.0–1.3)	0.095
October	1,500	262	(17.5)	1.1	(1.0–1.3)	1.1	(1.0–1.3)	0.126
Admission								
Outpatient	8,660	1,407	(16.3)	1.1	(1.0–1.2)	0.9	(0.7–1.2)	0.553
Inpatient	3,116	458	(14.7)	Reference		Reference		

^*^ Column percentage.

^†^ Includes Citobacter species, Burkholderia, coagulase-negative Staphylococci, Enterobacter, Providencia.

PR = prevalence ratio; CI = confidence interval; aPR = adjusted PR.

The samples from patients aged 45–59 years (aPR 1.5, 95% CI 1.3–1.7) and ⩾60 years (aPR 1.4, 95% CI 1.2–1.6) had significantly higher proportion of MDR than those aged 18–29 years (Table 6). The males (aPR 1.3, 95% CI 1.2–1.5) compared to females and in-patients (aPR 1.4, 95% CI 1.2–1.7) compared to outpatients had significantly higher proportions of MDR.

**TABLE 6:** Demographic characteristics associated with multidrug resistance among adult samples undergoing urine culture and drug susceptibility test in Kathmandu, Nepal, May–October 2019 (n =1,865)

Characteristics	Total *N*	MDR		PR	(95% CI)	aPR	(95% CI)	*P* value

		*n*	(%)^*^					
Total	1,865	1,000	(53.6)					
Age, years								
18–29	544	216	(39.7)	Reference		Reference		
30–44	420	217	(51.7)	1.3	(1.1–1.5)	1.2	(1.1–1.4)	0.007
45–59	349	226	(64.8)	1.6	(1.4–1.9)	1.5	(1.3–1.7)	<0.001
⩾60	552	341	(61.8)	1.6	(1.4–1.8)	1.4	(1.2–1.6)	<0.001
Sex								
Male	640	436	(68.1)	1.5	(1.4–1.6)	1.3	(1.2–1.5)	<0.001
Female	1,216	558	(45.9)	Reference		Reference		
Not recorded	9	6	(66.7)	1.5	(0.9–2.3)	1.2	(0.8–2.0)	0.365
Department								
Medicine	32	27	(84.4)	2.2	(1.6–3.1)	1.5	(1.1–2.1)	0.010
Surgery	127	96	(75.6)	2.0	(1.5–2.6)	1.5	(1.1–2.0)	0.006
Obstetrics/Gynaecology	81	31	(38.3)	Reference		Reference		
Nephrology	31	29	(93.6)	2.4	(1.8–3.3)	1.7	(1.2–2.3)	0.001
Others^†^	167	104	(62.3)	1.6	(1.2–2.2)	1.2	(0.9–1.7)	0.208
Not recorded	1,427	713	(50.0)	1.3	(1.0–1.7)	1.4	(1.0–2.0)	0.060
Month of referral								
May	274	164	(59.9)	Reference		Reference		
June	286	167	(58.4)	1.0	(0.8–1.1)	1.0	(0.9–1.1)	0.904
July	308	169	(54.9)	0.9	(0.8–1.1)	1.0	(0.8–1.1)	0.454
August	365	181	(49.6)	0.8	(0.7–1.0)	0.9	(0.7–1.0)	0.021
September	370	191	(51.6)	0.9	(0.8–1.0)	0.9	(0.8–1.0)	0.116
October	262	128	(48.9)	0.8	(0.7-1.0)	0.8	(0.7–1.0)	0.026
Admission								
Outpatient	1,407	1,163	(49.5)	Reference		Reference		
Inpatient	458	303	(66.2)	1.3	(1.2–1.5)	1.4	(1.2–1.7)	0.001

^*^ Column percentage;

^†^ Include Orthopaedics; Ear, Nose, Throat; Psychiatry; Burn Ward; Intensive Care Unit.

MDR = multidrug resistance; PR = prevalence ratio; CI = confidence interval; aPR = adjusted PR.

## DISCUSSION

This study reports on the prevalence of drug resistance among outpatient and inpatient urine samples being evaluated for possible UTIs in a referral hospital in Kathmandu, Nepal. The key findings include 1) the proportions of confirmed UTIs in outpatient and inpatient samples were respectively 16.2% and 14.7%; 2) the proportions of resistance to at least one antibiotic in outpatient and inpatient samples were respectively 82.7% and 89.5%; and 3) the proportions with MDR in outpatient and inpatient samples were respectively 49.5% and 66.2%.

The overall proportion of UTIs found was 15.8% in our study. In contrast, a study conducted in a similar teaching hospital in 2012 reported a prevalence of urine culture positivity of 32%.^20^ Kumar et. al reported a UTI prevalence of 25% among all urine samples tested.^21^ Although the reason for this difference is unclear, the decrease in the proportion could be due to population variances or increased screening practice, such testing for routine surgical procedures, asymptomatic bacteriuria, etc.

*E. coli* was the most frequent pathogen among outpatients (67.7%); inpatient UTIs were due to a more heterogeneous distribution of pathogens (*E. coli* 45%, *K. pneumoniae* 11%, *Enterococcus* 18% and *Pseudomonas* 15%). Similar to our findings, *E. coli* has been found to be the predominant pathogen by others.^2^,^17^,^20^,^22^

In our study, 84% of samples were resistant to at least one antibiotic and 54% were multidrug-resistant overall, which is of significant concern. Another study from Nepal in 2012 reported MDR in 41% of isolates.^23^ This suggests an increasing rate of MDR among urinary pathogens in Nepal, which should raise considerable alarm about the current state of antibiotic stewardship in the country.

When looking at specific pathogens and their level of resistance, we found several worrying findings. *E. coli* were highly resistant to advanced-generation antibiotics (ceftazidime 83%, levofloxacin 77% and cefepime 67%). In addition, *K. pneumoniae* were also significantly resistant (ceftazidime 96%, levofloxacin 80% and cefepime 83%). This high resistance to advanced-generation antibiotics is possibly because these drugs are tested for organisms which are found resistant to first-line drugs. Moreover, *Enterococcus* was highly resistant to some antibiotics (amoxicillin-clavulanate 43%, nitrofurantoin 27%), but not to vancomycin (2%). A review article from Nepal reported highest resistance of *E. coli* to amoxicillin, cefixime and amoxicillin-clavulanate.^17^ Our findings are consistent with another study showing alarmingly high resistance for fluoroquinolones and third-generation cephalosporins.^23^ A systematic review of studies from the Asia-Pacific region has reported a high prevalence of resistance of Gram-negative organisms to cotrimoxazole in Bangladesh (58%), Bhutan (53%) and India (64–74%), while a high prevalence was observed for ceftazidime.^13^ The drug resistance shown by *Enterococcus* with amoxicillin, nitrofurantoin and vancomycin were respectively 45%, 27% and 2%. This higher prevalence of drug resistance might be attributed to unnecessary prescription of antibiotics without bacterial confirmation or susceptibility testing, easy access to drugs (over-the-counter) and poor compliance to treatment.^24^,^25^

The only associated risk factor for infection in both outpatients and inpatients was age ⩾45 years (*P* < 0.001), which is in line with other results.^26^ Increased age and male sex were also associated with increased drug resistance in previous studies.^12^,^23^,^27^,^28^ Finally, inpatients were more likely to have MDR in our study. These findings might be attributed either to inpatient antibiotic practices and empirical therapy or failed empirical therapy among outpatients who might have ended up as inpatients — both are significant causes for concern.

### Strengths and limitations

A strength of our study was that it included all urine culture samples sent to the hospital laboratory during a 6-month period, which makes the findings generalisable to a similar setting. Also, we followed STROBE (Strengthening the Reporting of Observational Studies in Epidemiology) guidelines in reporting our study findings.^29^ Finally, the study was conducted in a large, referral, academic setting, where antibiotic stewardship should be a priority issue. Hence, this could provide guidance in the creation of a standard hospital treatment protocol. Possible study limitations include 1) the single-hospital setting, which might not represent the scenario of other hospitals, 2) no information on the annual trend due to the review of only 6 months of data, and 3) missing information on referring departments for outpatients and other clinical characteristics that might be associated with culture positivity and resistance, as the study was based on available hospital records. Finally, inpatient medical records could not be further examined to document treatment outcomes because of access limitations due to the Covid-19 pandemic.

These study results can provide valuable insights into the current state of AMR among urinary pathogens in TUTH and could provide guidance to hospital pharmacy and therapeutics personnel. Clear recommendations and actions regarding antimicrobial stewardship and guidance on specific treatment recommendations for UTI management could likely improve patient care and outcomes while reducing cost of care for both patients and the hospital.

Analysis of hospital data should be conducted routinely in order to facilitate generation of an antibiogram (an overall profile of antimicrobial susceptibility testing results of a specific micro-organism to a battery of antimicrobial drugs),^30^ which could be shared with clinicians for better understanding of AMR trends. In addition, our findings are likely to be similar to other tertiary care facilities in the region at this time. These results should alert other stakeholders, including policy makers and hospital directors regionally and perhaps nationally, to recognise the rising challenge of AMR in both outpatient and inpatient settings. There is a need to develop more routine surveillance nationwide, which could lead to strategies for preventing further bacterial resistance.^24^,^31^ Government policies should also address restrictions on access to antibiotics and social awareness on compliance.^24^

There is clearly a need to conduct similar studies, over a greater length of time and in other settings throughout Nepal to confirm these findings. Our hope is that we can avoid further escalation of the AMR crisis, which would have a significant impact upon patient outcomes and the economy of Nepal.

## CONCLUSION

In a large academic referral hospital in Kathmandu, Nepal, we found a rising proportion of MDR UTIs than has previously been reported, especially within the inpatient setting. Support for improved antibiotic stewardship and enhanced treatment guidance for UTIs is recommended to reverse this course. These findings are likely similar in comparable tertiary care facilities in the region, but further multi-centric studies need to be conducted to confirm this.

## References

[1] World Health Organisation (2011). Urinary tract infection.

[2] Gupta P, Gupta K (2018). The profile of uropathogens and their antibiotic susceptibility in IPD adults in a tertiary care hospital in North India. Int J Curr Microbiol App Sci.

[3] Stamm WE, Norrby SR (2001). Urinary tract infections: disease panorama and challenges. J Infect Dis.

[4] World Health Organisation (2014). Antimicrobial resistance global report on surveillance, 2014.

[5] Tancharoensathien V, Chanvatika S, Sommanustweechai A (2018). Complex determinants of inappropriate use of antibiotics. Bull World Heal Organ.

[6] Shakya P (2017). ESBL Production Among *E. coli* and *Klebsiella* spp. Causing urinary tract infection: a hospital based study. Open Microbiol J.

[7] Dickstein Y (2016). Predicting antibiotic resistance in urinary tract infection patients with prior urine cultures. Antimicrob Agents Chemother.

[8] World Health Organisation (2017). Global action plan on antimicrobial resistance.

[9] Rai GK (2008). Causative agents of urinary tract infections in children and their antibiotic sensitivity pattern: a hospital based study. Nepal Med Coll J.

[10] Khan MS (2019). LMICs as reservoirs of AMR’: a comparative analysis of policy discourse on antimicrobial resistance with reference to Pakistan. Health Policy Plan.

[11] Raka L (2019). Point prevalence survey of healthcare-associated infections and antimicrobial use in Kosovo hospitals. Infect Dis Rep.

[12] Gomila A (2018). Predictive factors for multidrug-resistant gram-negative bacteria among hospitalised patients with complicated urinary tract infections. Antimicrob Resist Infect Control.

[13] Sugianli AK (2021). Antimicrobial resistance among uropathogens in the Asia-Pacific region: a systematic review. JAC Antimicrobial Resist.

[14] Ganesh R (2019). Epidemiology of urinary tract infection and antimicrobial resistance in a pediatric hospital in Nepal. BMC Infect Dis.

[15] United Nations, Department of Economic and Social Affairs (2021). Sustainable development.

[16] Zellweger M (2017). A current perspective on antimicrobial resistance in Southeast Asia. J Antimicrob Chemother.

[17] Basnyat B (2015). Antibiotic use, its resistance in Nepal and recommendations for action: a situation analysis. J Nepal Health Res Counc.

[18] Clinical Laboratory Standards Institute (2015). Performance standards for antimicrobial disk susceptibility tests: approved standard.

[19] Magiorakos AP (2012). Multidrug-resistant, extensively drug-resistant and pan-drug-resistant bacteria: an international expert proposal for interim standard definitions for acquired resistance. Clin Microbiol Infect Dis.

[20] Rijal A (2012). Antibiotic susceptibility of organisms causing urinary tract infection in patients presenting to a teaching hospital. J Nepal Health Res Counc.

[21] Kumar A (2017). Antimicrobial susceptibility pattern of urine culture isolates in a tertiary care hospital of Jharkhand, India. Int J Basic Clin Pharmacol Orig Res Artic.

[22] Medina M, Castillo-Pino E (2019). An introduction to the epidemiology and burden of urinary tract infections. Ther Adv Urol.

[23] Baral P (2012). High prevalence of multidrug resistance in bacterial uropathogens from Kathmandu, Nepal. BMC Res Notes.

[24] Adhikari S (2019). Emergence of antimicrobial drug resistant bacteria in Nepal: a current scenario. J Proteomics Bioinform.

[25] Acharya KP, Wilson RT (2019). Antimicrobial resistance in Nepal. Front Med.

[26] Schmiemann G (2010). The diagnosis of urinary tract infection: a systematic review. Dtsch Arztebl Int.

[27] Karlowsky JA (2011). Antimicrobial resistance in urinary tract pathogens in Canada from 2007 to 2009: CANWARD surveillance study. Antimicrob Agents Chemother.

[28] Tenney J (2018). Risk factors for aquiring multidrug-resistant organisms in uri-nary tract infections: a systematic literature review. Saudi Pharm J.

[29] Gharaibeh A, Koppikar SJ, Bonilla-Escobar F (2014). Strengthening the Reporting of Observational Studies in Epidemiology (STROBE) in the International Journal of Medical Students. Int J Med Students.

[30] Minnesota Department of Health, Infectious Disease Epidemiology, Prevention, and Control Division (2015). About antibiograms.

[31] Dahal RH, Chaudhary DK (2018). Microbial infections and antimicrobial resistance in Nepal: current trends and recommendations. Open Microbiol J.

